# Serological surveillance of *Leucocytozoon* spp. in Taiwan

**DOI:** 10.1016/j.psj.2025.105729

**Published:** 2025-08-24

**Authors:** Yen-Cheng Lin, Chih-Feng Chen, Akira Ito, Naomi Himeno, Tzu-En Lin

**Affiliations:** a Department of Animal Science, National Chung Hsing University, Taichung 402, Taiwan; b The iEGG and Animal Biotechnology Center, National Chung Hsing University, Taichung 402, Taiwan; c Hokkaido Green Pharma Corporation, Japan; d Graduate Institute of Biomedical Sciences, China Medical University, Taichung 404, Taiwan

**Keywords:** ELISA, Serology, *Leucocytozoon*, rR7 antigen, Taiwan

## Abstract

Chicken Leucocytozoonosis is a protozoan disease that affects the blood and tissues of chickens. High summer temperatures create optimal conditions for reproducing biting midges, the primary vectors for transmitting *Leucocytozoon* to chickens. In Taiwan, a subtropical region, the disease remains prevalent throughout the year, except in winter, leading to significant economic losses due to increased mortality and reduced egg production. Despite its impact, diagnosing this disease remains challenging. While clinical symptoms may suggest infection, rapid serological diagnostic methods are essential for laboratory confirmation and effective disease management. This study used the recombinant R7 (rR7) antigen to measure enzyme-linked immunosorbent assay (ELISA) antibody titers. Blood samples from 260 chickens, including laying and breeding hens, were collected from Central and Southern Taiwan between August and October 2024. Both polymerase chain reaction (PCR) antigen and ELISA antibody tests were conducted from the blood. Clinically, the 23 tested chicken flocks exhibited common symptoms, including noticeable greenish diarrhea, reduced egg production, cracked- and soft-shelled eggs, and pale comb of anemia characterized by pale combs. The results revealed that the ELISA antibody test showed a 33.5 % positive infection rate, while the PCR test showed only 0.43 %. Thus, ELISA antibody testing proves to be a sufficient diagnostic method in the early infection stage. Because of the high infection rate of *Leucocytozoon* in Taiwan, this study highlights a renewed understanding of the prevalence of Lecocytozoonosis over the past three decades. It also emphasizes the urgency of introducing the vaccines in Taiwan.

## Introduction

Leucocytozoonosis is a parasitic disease affecting a wide range of wild and domestic avian species. Transmitted by the biting midge, *Culicoides arakawae* (***C. arakawae***), the disease is caused by the protozoan parasite, *Leucocytozoon* spp. (***L.* spp.**), which infects endothelial cells of microvasculature, cells, as well as plasma and red blood cells, in chickens. Within the genus *Leucocytozoon*, the protozoan *Leucocytozoon caulleryi* poses a particularly severe threat to chickens. This discussion will therefore focus on this specific parasite. The parasite's complex 28-day life cycle alternates between chicken hosts and midge vectors, leading to disease manifestation following parasitic reproduction within both insects and chickens (Inoue, 1989; Nakamura et al., 2001). Leucocytozoonosis results in substantial economic losses due to elevated mortality rates, diminished egg production, and post-infection sequelae (Elbestawy, et al., 2021; Lee, 1968; Nakamura, Ogiso, Shibahara, Kasuga and Isobe, 2001; Yu, et al., 2000). Similar to malaria in humans, the incidence of Leucocytozoonosis peaks in the summer, while it is relatively low during the winter.

The environmental conditions suitable for the vector, *C. arakawae*, are often found in the vicinity of rice paddies and overgrown weeds during warm seasons, particularly in summer. (Kitaoka and Morii, 1963). Temperature and humidity are the primary environmental parameters that create conditions suitable for the vector. In Taiwan and other Southeast Asian countries, there are two or three rice cropping seasons, thus creating prolonged periods of standing water in paddies, which support the massive reproduction of midges (Yu, Wang and Yeh, 2000). Since most Taiwanese layer farms adopt an open housing system, midges can easily invade, leading to a peak in disease incidence for 7 to 8 months each year (from March to October) (Kitaoka and Morii, 1963). However, unlike what might be expected, the peak does not occur in the hottest months (July and August), but rather during the cooler spring and autumn months (March to April and October to November), when temperatures are between 25 and 28 °C (Akiba, 1970; Yu, Wang and Yeh, 2000).

Most Taiwanese layer farms are located near rice paddies, which increases the risk of midge infestation. In addition to midge control, more proactive disease prevention measures are necessary (Yu, Wang and Yeh, 2000). Although parasitemia was observed only from 14 to 24 days after sporozoite inoculation in SPF chickens, ELISA antibodies against schizonts of *L. caulleryi* were detected in experimentally infected chickens for an extended period of 2-50 weeks (Isobe and Suzuki, 1987). Furthermore, in naturally infected chickens that survived, schizonts were found in various internal organs such as brains, livers, and spleens over two summer seasons (Inoue, 1989). The presence of schizonts in chickens provides a source for *Leucocytozoon* infection routes in subsequent summer seasons. Previous investigations have documented the prevalence of leucocytozoonosis in Taiwan for decades. Early research, such as a 1958 survey, reported a natural infection rate of 9 % and a mortality rate of 30 % in adult chickens, while chicks showed a higher infection rate of 22 % but a lower mortality rate of 15 % (Lee, 1968). Subsequent serological studies from 1971 to 1973 on samples from Miaoli, Taichung, and Kaohsiung further revealed a high seroprevalence (antibody-positive rate), ranging from 41.2 % to 93.8 %, indicating widespread exposure to the disease. The antigenaemia rate (antigen-positive rate), however, was lower, ranging from 0 % to 20 % during this period. The endemic nature of the disease was ultimately confirmed by a year-round serological survey conducted by Morii et al. (1981), which demonstrated that *Leucocytozoon* infection is both widespread and persistent throughout the year in Taiwan's chicken population (Morii, et al., 1981). Current serological methods for monitoring the disease, such as polymerase chain reaction (**PCR**) (Chiang, et al., 2022; Imura, et al., 2012; Zhao, et al., 2016), blood morphological examinations, and agar gel precipitation (Isobe, et al., 1998), can only detect infections in the later stages (14 days after infection) and cannot predict the prevalence of the disease in their early stages. Additionally, the recombinant R7 (**rR7**) antigen, previously used as a vaccine antigen for Leucocytozoonosis in Japan, is derived from the second schizont of *L. caulleryi* and expressed in *E. coli* (Itoh and Gotanda, 2002). An assay with this rR7 antigen was reported to detect *L. caulleryi* infection in its early stages (Ito and Gotanda, 2005). Therefore, it is crucial to promote the widespread use of the rR7 antigen in serological testing and to fully understand the losses caused by infections in layer farms to implement timely and effective preventive measures in Taiwan.

This study aimed to evaluate the sensitivity of two molecular techniques, PCR and enzyme-linked immunosorbent assay (**ELISA**), for the detection of Leucocytozoonosis in chickens, where the ELISA utilized the rR7 antigen developed by Ito and Gotanda, which has been shown to have high specificity for *L. caulleryi* and can detect antibody seroconversion as early as 7-13 days post-infection (Ito and Gotanda, 2005). The PCR method employed in this study is the same as the method previously described by Chiang et al. in 2022 (Chiang, Lin, Wang, Lee and Chen, 2022). Their study validated the high sensitivity and accuracy of this PCR approach. This validation demonstrated the capacity of the method to detect the target gene even when the prevalence of infection is low. Furthermore, it illustrated the ability to identify the gene even at low levels of infection within individual hosts. Notably, the limit of detection for this PCR method is approximately 2 infected blood cells within a sample of 50 million red blood cells (Zhao, Pang, Xu, Liu, Liu, Li and Su, 2016).

## Materials and methods

### Blood samplings

Blood samples were collected from chickens across nine farms in Central and Southern Taiwan between August and October 2024. The sampling method primarily involved farm-level observation with random sampling conducted after clinical signs were observed; importantly, not all sampled chickens exhibited clinical signs, so their infection status remained unknown. The samples were coagulated and centrifuged at 600 × *g* for 10 min at room temperature. The resulting sera and blood clots were preserved for ELISA and PCR testing, respectively.

### Farm distribution and clinical observations in chickens

Nine farms (HO, WN, C, Chan, S, Zhen, Zhong, W, and L) and 23 chicken flocks in Taiwan were selected for blood sampling. Blood samples were collected from 260 chickens, which included 200 laying hens and 60 breeder hens, with the ages of the birds ranging from 133 to 686 days. Most chickens were in the laying phase, having egg production rates ranging from 20 to 70 %. Clinical symptoms observed included decreased egg production, cracked- and soft-shelled eggs, and greenish diarrhea. Affected chickens also showed clinical signs of marked anemia and lethargy. The farms were situated in environments adjacent to rice paddies or overgrown weeds, which provided favorable breeding grounds for biting midges and other insects.

### DNA extraction

DNA samples were extracted from chicken blood clots using the Biokit Blood or Tissue and Cell Genomic DNA Purification Kit (Biokit, Taiwan). The extracted DNA samples were stored at −20 °C until further analysis.

### Blood smears

A 2 µL blood sample was placed on a glass slide. Then, a blood smear is prepared by a second slide. After that, the blood smears were stained with a BASO Wright-Giemsa staining kit (BASO Biotech, Zhuhai, China) for 5 min. The stained slides were observed for *Leucocytozoon* gametocytes was examination under a microscope at 200 × and 400 × magnification, with particular attention paid to the edges. The key diagnostic feature for *L. caulleryi* infection is the presence of large macrogametocytes, which typically appear oval or round. These parasites cause the host erythrocytes to swell or become distorted, leading to a displaced or absent host cell nucleus. This morphological anomaly is a critical characteristic for identifying the infection.

### Polymerase chain reaction assay

To detect *L. caulleryi* in the chicken blood, we amplified cytochrome *b* (***cyt-b***) gene fragments of the parasite with a pair of primers designed based on the mitochondrial DNA sequence of *L. sabrazesi.* These primers have been previously validated to also detect *L. caulleryi* in chicken blood, despite being based on the mitochondrial DNA of a different species (Chiang, Lin, Wang, Lee and Chen, 2022; Zhao, Pang, Xu, Liu, Liu, Li and Su, 2016). PCR was conducted using a total volume of 10 µL containing 0.5 μM of each forward and reverse primer, 1 µL genomic DNA (30 to 50 ng), 3 µL sterilized distilled water, and 5 µL Dream Taq Green PCR Master Mix (2 ×; Thermo Scientific, Waltham, MA). We amplified the chicken *cyt-b* gene fragments to serve as an internal control for successful extraction of DNA. Additionally, we included both positive and negative controls to confirm the validity and reliability of the PCR assay itself. The primer sequences and PCR conditions are listed in Table 1. PCR products were checked with 1.8 % agarose gel electrophoresis.

**Table 1:** Primer sequence and conditions of polymerase chain reaction.

Gene	Primer^a)^	Primer sequence (5′ to 3′)	PCR conditions^b)^	Production size (bp)
*G. gullaus Cyt b*	Gcytb_F	TAGTAGAGTGAGCCTGAGGG	95.0 °C 120sec 95.0 °C 30 sec, 63.0 °C 30 sec, 72.0 °C 60 sec, 72.0 °C 600 sec	231
	Gcytb_R	GGCTAGTGTTAGGAATGGGGTG		
*L. caulleryi Cyt b*	Lcytb_F	TAGTTTCATGGATATGTGGT	95.0 °C 120 sec, 95.0 °C 30 sec, 58.0 °C 30 sec, 72.0 °C 60 sec, 72.0 °C 600 sec	236
	Lcytb_R	ACTTTGTGATAAGAATAGTACT		

a) F, forward; R, reverse.

b) bold PCR step with 35 cycles.

### Enzyme-linked immunosorbent assay

An indirect ELISA for the detection of *L. caulleryi* antibodies was developed using purified rR7 antigen, as described in previous publications. The rR7 antigen was produced in *Escherichia coli* using the pMAL expression system (New England BioLabs, Massachusetts, USA). Following the expression, the antigen was purified by ion-exchange chromatography with DEAE-Sephacel (Pharmacia, Uppsala, Sweden) and gel chromatography with Superdex 200 (Pharmacia). The purified rR7 antigen was dialyzed against phosphate-buffered saline (PBS), its protein concentration was determined using a BCA Protein Assay Kit (Pierce Chemical, Illinois, USA), and it was then stored at −20 °C (Ito and Gotanda, 2005; Itoh and Gotanda, 2002; Suprihati, et al., 2025). Negative and positive controls were prepared using serum from SPF chickens and chickens immunized with rR7 antigen, respectively, and were diluted in the same manner. The 96-well microtiter plates were coated with the diluted rR7 antigen (100µL/well) and incubated overnight at 4 °C. After washing with PBST (phosphate-buffered saline containing 0.05 % Tween20), the plates were blocked with 200µL/well of ChonBlock ELISA buffer (Chondrex, Redmond, WA, USA) for 1 h at 37 °C. Samples were diluted 1:400 in a sample dilution buffer (20 % ChonBlock ELISA buffer in PBST). The plate was then incubated for 1 h at 37 °C before being washed. The secondary antibody, HRP-labeled goat anti-chicken IgY (*H* + *L*) (Thermo Fisher Scientific, Delaware, DE, USA), was diluted 1:20,000 in a similar customized dilution buffer (20 % ChonBlock detection antibody dilution buffer in PBST) and added for a 1-hour incubation at 37 °C. After final washes, TMB substrate (LGC Clinical Diagnostics, Teddington, UK) was added, and the reaction was stopped with 1mol/L hydrochloric acid after 20 min. The optical density (OD) was measured at 450 nm. A sample was considered positive if its sample-to-positive (S/P) ratio was equal to or greater than 0.3, while a ratio below 0.3 was interpreted as negative.

## Results

**Fig. 1:**
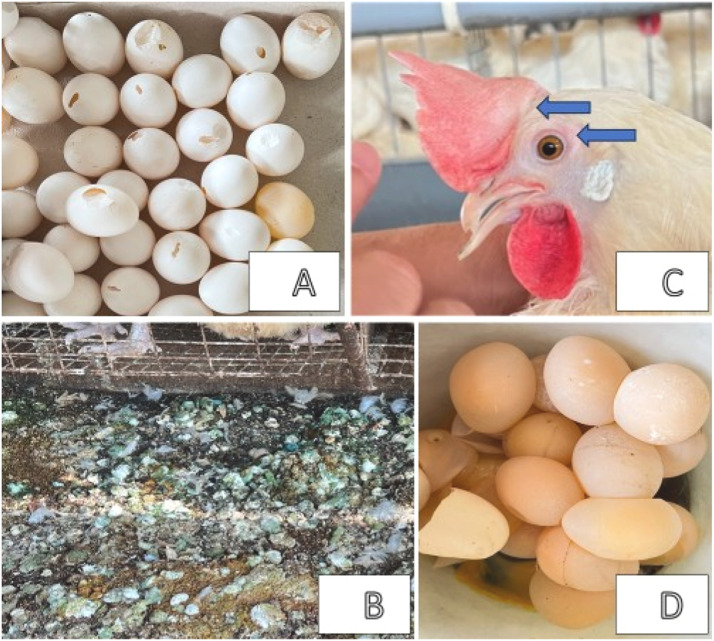
A. Cracked-shelled eggs by uneven calcium precipitation. B. Greenish diarrheal droppings on the floor. C. Pale comb and eye frame (arrows). D. Soft-shelled eggs.

In this study we assessed 23 chicken flocks, each consisting of 2,000 to 10,000 birds. Within each flock, 4 to 20 chickens were randomly selected for sampling (Table 2), leading to a total of 260 collected blood samples. All flocks displayed common clinical symptoms, including pronounced greenish diarrhea, decreased egg production, cracked- and soft-shelled eggs, and pale combs related to anemia, as depicted in Fig. 1A–D. For breeder chickens, in addition to the clinical symptoms observed in laying hens, the fertility rate of breeder eggs ranged from 50 % to 56 %. Hatching and survival rates for produced chicks were 40 and 30 % for the breeder chickens, respectively, in the C-1 flock. The surrounding environments of the chicken farms shared common characteristics. Farms located in flats were typically adjacent to rice paddy fields with overgrown weeds, while dense forests and extensive weeds surrounded those in hilly areas. As a result, biting midges (*C. arakawae)* were frequently observed swarming around the farms during the evenings, causing irritation and biting the chickens.

**Table 2:** Epidemiological survey of *L. caulleryi* in chickens in Taiwan by using ELISA assay of the rR7 antigen and PCR assay.

Farms and flocks for serum collecting	Location and environment			No. of birds tested (bird type) ^c^	Age in days	No. (and %) of positive	
	Central Taiwan^a^	Southern Taiwan	Environ-ment ^b^			ELISA assay	PCR^d^ assay
HO-1	Y		R	4(L)	133	0(0)	0(0)
HO-2	Y		R	14(L)	441	3(21.4)	0(0)
HO-3	Y		R	14(L)	238	1(7.1)	0(0)
HO-4	Y		R	18(L)	630	5(27.8)	0(0)
WN-1		Y	R	10(L)	560	3(30)	ND
WN-2		Y	R	10(L)	210	4(40)	ND
WN-3		Y	R	10(L)	560	0(0)	ND
C-1	Y		R	10(L)	565	3(30)	0(0)
Chan-1	Y		R	10(B)	374	2(20)	0(0)
S-1	Y		R	10(B)	287	9(90)	0(0)
Zhen-1	Y		R	20(B)	169	9(45)	0(0)
Zhong-1	Y		O	20(B)	252	6(30)	0(0)
W-1	Y		O	10(L)	311	7(70)	0(0)
W-2	Y		O	10(L)	368	8(80)	0(0)
W-3	Y		O	10(L)	562	8(80)	0(0)
W-4	Y		O	10(L)	479	8(80)	0(0)
L-1	Y		R	10(L)	227	0(0)	0(0)
L-2	Y		R	10(L)	686	3(30)	0(0)
L-3	Y		R	10(L)	137	0(0)	0(0)
L-4	Y		R	10(L)	747	2(20)	0(0)
L-5	Y		R	10(L)	594	5(50)	1(10)
L-6	Y		R	10(L)	514	1(10)	0(0)
L-7	Y		R	10(L)	172	0(0)	0(0)
Total				260		87(33.5)	1(0.43)

a) Farm location: ST denotes Southern Taiwan, and CT denotes Central Taiwan.

b) Adjacent environment: R indicates rice paddies; O indicates overgrown weeds.

c) Bird type: B denotes Breeders; L denotes Layers.

Number of samples tested: 230; ND (Not Done) for 30 samples from farms WN-1 to WN-3.

The sampling of chickens was conducted in two phases in two different seasons. The first phase occurred in summer (August) in Taiwan, with temperatures in Central and Southern Taiwan ranging from 29 to 33 °C and 29 to 35 °C, respectively. The second phase was conducted during the autumn season (October to November), with temperatures in Central and Southern Taiwan ranging from 22 to 27 °C and 22 to 29 °C, respectively. Both seasons provided ideal breeding conditions for biting midges. The sampling environments included farms located near rice paddies or overgrown weeds in Central or Southern Taiwan (Table 2).

The analysis of blood smears obtained from all sampled birds yielded negative results for parasites. PCR analysis of 230 chickens from 20 flocks (excluding WN-1, WN-2, and WN-3) detected only one positive case (0.43 %) in flock l-5, with all other samples testing negative for *Leucocytozoon* (Table 2). In contrast, ELISA results indicated a predominantly higher prevalence with 87 positive cases (33.5 %) detected among all 260 chickens including PCR-positive sample. Positive cases were found in flocks: HO-2 (3/14, 21.4 %), HO-3 (1/14, 7.1 %), HO-4 (5/18, 27.8 %), WN-1 (3/10, 30 %), WN-2 (4/10, 40 %), C-1 (3/10, 30 %), Chan-1 (2/10, 20 %), S-1 (9/10, 90 %), Zhen-1 (9/20, 45 %), Zhong-1 (6/20, 30 %), W-1 (7/10, 70 %), W-2 (8/10, 80 %), W-3 (8/10, 80 %), W-4 (8/10, 80 %), l-2 (3/10, 30 %), l-4 (2/10, 20 %), l-5 (5/10, 50 %), and l-6 (1/10, 10 %).

Among the 23 flocks used in the trial, 18 (78.3 %) exhibited positive cases by ELISA. Positive cases were detected in three out of four flocks in the HO farm, two out of three flocks in the WN farm, all four flocks in the W farm, and four out of seven flocks in the L farm. Additionally, the five farms (C, Chan, S, Zhen, and Zhong) with only one flock all tested positive. When analyzed by bird type, 63 out of 200 chickens (31.5 %) from 14 out of 19 layer flocks tested positive. In contrast, 24 out of 60 chickens (40 %) from all four breeder flocks were positive. Further stratification by age groups (<6months, 7-12months, 13-18 months, 19-24 months, >24 months) revealed positivity rates of 9 cases (20.4 %), 27 cases (36.4 %), 22 cases (40.7 %), 0 cases (0 %), and 29 cases (32.9 %), respectively.

## Discussions

Southeast Asian countries including Taiwan, are located in the subtropical to tropical regions with temperatures ranging from 25 to 35 °C. This region provides optimal conditions for the proliferation of biting midges (Yu, Wang and Yeh, 2000). These conditions facilitate the transmission of *Leucocytozoon* to chickens through bites from infected midges, leading to infections characterized by clinical symptoms such as mortality, decreased egg production, greenish diarrhea, and soft-shelled eggs (Akiba, 1970; Nakamura, et al., 1997). Among many infectious diseases affecting chickens, reduced egg production is a prevalent symptom in both laying and breeding chickens. While this symptom is often attributed to viral (Belser, et al., 2013), and bacterial infections, the occurrence of cracked- and soft-shelled eggs along with greenish diarrhea is particularly indicative of *Leucocytozoon*, as shown in the figures (Fig. 1A–C). Consequently, the development of a reliable and accurate method for early diagnosis is urgently needed. The peak season for Leucocytozoonosis in Taiwan spans from April to October. In the present study, we focused on the period from late summer to early autumn, specifically August to October. Blood samples were randomly collected from a total of 260 birds from 23 flocks (19 layer and 4 breeder flocks) in Central and Southern Taiwan. Farmers from these farms reported decreased egg production, greenish diarrhea, and cracked- and soft-shelled eggs, which have a significant impact on the poultry industry. The samples were analyzed using ELISA and PCR antibody tests to compare their effectiveness in providing accurate serological diagnoses. However, previous research efforts have failed to confirm infections using laboratory data, such as blood smears or PCR tests (Chiang, Lin, Wang, Lee and Chen, 2022; Zhao, Pang, Xu, Liu, Liu, Li and Su, 2016). This study aimed to develop a simple solution to address these symptoms and conduct an epidemiological investigation of Leucocytozoonosis under various environmental conditions in Taiwan.

The ELISA employed in this study contains rR7 protein from *L. caulleryi* as the antigen (Gotanda, et al., 2002; Ito and Gotanda, 2004, 2005; Itoh and Gotanda, 2002). The efficacy of the rR7 antigen, derived from the second-generation schizonts of *L. caulleryi*, has been verified under field conditions for detecting chicken Leucocytozoonosis(Ito and Gotanda, 2005). The detected infection positivity rate of 33.5 % suggests that *L. caulleryi* is a major contributor to the prevalence of Leucocytozoonosis in Taiwan. While the anti-parasitic properties of *Artemisia annua* have been shown to be prioritized as a primary preventive strategy in Taiwan(Chiang, Lin, Wang, Lee and Chen, 2022), the rR7 ELISA has demonstrated its capacity to assess the losses associated with *L. caulleryi* infections. However, there remains an urgent need to develop vaccines utilizing rR7 as the antigen to prevent pale comb disease caused by *L. caulleryi* strain effectively.

In the present study, we conducted large-scale blood sampling during late summer and early autumn (from August to October) under the optimal climatic conditions for the proliferation of biting midges in Taiwan. To the best of our knowledge, this is the first time we have examined *L. caulleryi* infections in chickens from Miaoli, Changhua, Nantou, Yunlin, Pingtung counties, and Taichung City using ELISA. Samples were collected from barns exhibiting clinical observations such as the presence of greenish diarrhea, cracked- and soft-shelled eggs, and pale comb. Results indicated that a total of 87 out of 260 chickens (33.5 %) were confirmed infected with *L. caulleryi*. The prevalence rates by region were 77.5 % (31/40) in Miaoli County, 17.8 % (16/90) in Changhua County, 30.0 % (6/20) in Nantou County, 60 % (18/30) in Yunlin County, 23.3 % (7/30) in Pingtung County, and 18.0 % (9/50) in Taichung City.

The microscopic examination determined that none of the 260 samples were positive in blood smears. In contrast, PCR detection revealed that only 1 out of 230 samples (0.4 %) was positive. The lower detection rate of PCR compared to previous serological surveys can be attributed to the limited time window for PCR-based diagnosis. The PCR method is most effective at detecting parasite DNA during a specific phase of the *L. caulleryi* life cycle, typically between days 14 and 21 post-infection. Consequently, this method may fail to identify chickens that are either uninfected or have already been infected but are outside this specific diagnostic window. Therefore, ELISA is approved to be a more sensitive method for identifying positive samples. While Morii T. et al. detected antibodies in the blood only 17 days after experimentally infecting chickens (Morii, et al., 1986), Isobe T. *et al*. demonstrated that ELISA could detect early antibodies as early as 14days post-infection (Isobe and Suzuki, 1987). In this field study, positive ELISA antibodies were detected earlier than those determined by PCR.

This study underscores the limitations of traditional blood smear microscopy for diagnosing Leucocytozoonosis, particularly regarding its low sensitivity during early life cycle stages or in cases of low parasitemia, which can pose challenges for less experienced personnel (Chatan, et al., 2024; Vaisusuk, et al., 2022). Recent advancements have led to the increased adoption of molecular methods in laboratories, with PCR demonstrating high specificity and sensitivity in detecting *L. caulleryi* infection, even with low parasitemia or during the early stages of infection (14 days post-infection, dpi). However, ELISA examination of serum samples is much more sensitive and detects a greater number of positive cases. Previous research has shown ELISA's ability to detect infections at earlier stages (7 dpi) and even weeks after apparent recovery (Ito and Gotanda, 2005). Therefore, ELISA is a more suitable method for detecting *L. caulleryi* infections, particularly for monitoring of epidemiological trends of the disease.

The ELISA used in this study employs the rR7 antigen of the *L. caulleryi* strain, which has historically been the primary pathogenic strain in Taiwan (Ito and Gotanda, 2005; Morii, Nakamura, Lee, Iijima and Hoji, 1986). Using the rR7 antigen of the *L. caulleryi* strain, this study detected a 33.5 % infection positivity rate, indicating that *L. caulleryi* is currently the leading cause of the prevalence of Leucocytozoonosis in Taiwan. In the future, it is essential to base vaccine prevention measures for Leucocytozoonosis on the *L. caulleryi* strain and establish effective prevention strategies for chickens against this disease.

Accurately identifying specific infectious diseases affecting laying hens that exhibit symptoms, such as decreased egg production, is often challenging, leading to frequent misdiagnoses. This study demonstrates that the application of ELISA for the surveillance of Leucocytozoonosis can significantly aid in the rapid detection and evaluation of vaccine efficacy, particularly in Taiwan and other Southeast Asian countries with hot climates. Additionally, the antibody detection using the method of immunoblot was compared with the ELISA or AGP methods by Isobe, T. et al., indicating that both methods effectively confirmed the presence of antibodies and identified antibody-positive chickens as a response to infection (Isobe, Shimizu, Yoshihara and Suzuki, 1998).

Although *Artemisia annua*, the antimalarial herb, was proven to be effective against *Leucocytozoon*, vaccination measures should be considered as a main preventive strategy for the poultry industry in Taiwan (Chiang, Lin, Wang, Lee and Chen, 2022). The application of the rR7 ELISA in this study has provided a comprehensive understanding of the mortality rates and economic losses caused by *L. caulleryi* infections in Taiwan. Therefore, it is mostly urgent to introduce the vaccine of rR7 antigens for effective preventive measures (Ito and Gotanda, 2004; Umali, et al., 2014).

In conclusion, these findings contribute significantly to our understanding of the epidemiology of Leucocytozoonosis in Taiwan. The evaluation of the efficacy of ELISA highlights its potential as a valuable tool for monitoring disease trends and assessing the effectiveness of control measures.

## CRediT authorship contribution statement

**Yen-Cheng Lin:** Writing – original draft, Investigation, Formal analysis, Data curation. **Chih-Feng Chen:** Supervision, Resources, Project administration, Funding acquisition. **Akira Ito:** Writing – review & editing, Supervision, Resources, Methodology. **Naomi Himeno:** Writing – review & editing, Resources. **Tzu-En Lin:** Writing – original draft, Supervision, Project administration, Methodology, Investigation, Data curation, Conceptualization.

## Disclosures

The authors have no conflicts of interest directly related to the content of this article.
